# Investigations of the Tooth Pulp

**Published:** 1871-03

**Authors:** Franz. Boll

**Affiliations:** Berlin, Prussia


### DENTAL REGISTER.

VOL. XXV.] MARCH, 1871. [No. 3.

### Communications.

### INVESTIGATIONS OF THE TOOTH PULP.

BY DR. FRANZ. BOLL, OF BERLIN, PRUSSIA.

Translated by Henriette Hirschfeld, D. D. S.

BY S. P. CUTLER, M. D., D. D. S.

At the time of these investigations, the author was a medical student, since assistant to DuBois Raymond, Prof. of Physiology in the University of Berlin.

Prof. Waldeyer, of the University of Breslau, eminent as a histologist and microscopist, says, in this splendid essay on the Mouth and Development of the Teeth, "we owe to Mr. Boll the first definite knowledge of the condition of the nerve fibres in the teeth."

The writer says, "In the histology of tooth tissue, I have directed my attention mainly to two points, the one being slightly the other scarcely at all studied." I allude to the termination of the many nerve fibres entering into each tooth. *This fact I pointed out long ago.*

"The second, which has already produced a very extensive literature, treats of the propositions of the fundamental tooth substance, the tooth pulps, and the origin of the first out of the latter."

1st. "The Termination of the Nerve Fibres. For the investigation of the nerve termination, I can recommend as most suitable, the long incisors of rodents, guinea pigs, rabbits, etc."

Here the author falls into the same error with Beale in calling the teeth of rodents incisors. These teeth are cuspids instead of incisors, though occupying the position of incisors of other animals.

His method of preparing teeth is as follows: "If we take the incisor of an animal recently killed, crack it in a vise, extract the pulp, and apply a magnifying power of 200 diameters, it will be difficult to distinguish the coarser anatomical features." "The ramifications of a very rich network of vessels is to be seen consisting in part of capillaries, and in part of more substantial vascular branches, with a layer of non striated muscular fibres, and a large number of thick dark bordered nerve fibres, ascending parallel with the longitudinal axis of the pulp, in fasciculi of six or eight or more nerve fibrils." The non-striated muscular fibres spoken of, seems to me to be a miscomprehension of their true character, as I can see no use of any such fibres in the pulp, as muscular fibres presupposes the office or function of contractility of such fibres, and that in a tissue where there can not be any possible need or use of any such contractile fibres, any more than there would be in the marrow of the bones themselves. The author speaks of delicate connective tissue fibres, which are easily recognized from the baccated nerve fibres of the pulp.

But as to there being two distinct sets of fibres, distinct and separate from nerve fibres, I have yet to see that the connective tissue fibres are readily recognized, etc., as the author says, also a cellular tissue filling up all interspaces amongst nerve fibrils.

What office or function could muscular fibres perform in a tooth pulp, as the entire function of the pulp can be nothing more or less than that of sensation and nutrition, every sentient fibril being its own factor, capable at all times of transmitting the morbid function of pain to the brain, when a cause exists.

The author's method of preparing pulps for examination, is very ingenious and important as far as it goes. If we admit the existence of muscular fibres, we must admit the existence of moto nutrient nerves, otherwise no muscular functions could take place.

It has been generally conceived that the motor nerves mainly perform the function of motion, but it certainly would be more reasonable to allow every nerve, except perhaps, it be the nerves of the five senses, to furnish nutrition to the part supplied only by them. I am not quite certain even that there is any exception at all, as nutrition takes place in cartilage, where there are neither nerves nor blood-vessels.

The author speaks of the embryonical pulp, remaining not used in this formation, *meaning, no doubt, the formation of dentine*, as having lost their spider or spindle shaped form. Right here I must beg the privilege of differing with him in this respect, that the collaginous or cartilaginous tissues that are transformed into bones, in general never have the spider shaped form mentioned, this spider shaped form being always peculiar to bones, and are the effect of ossification, hence is secondary formation or process. The author here presupposes the existence of the canaliculi and lacunæ in the soft pulp, and then their subsequent disappearance.

In primary bone cartilage there is at first no blood vessels or nerves, the only recognizable structure under the microscope being a cell structure, and an intermediate homogenious substance, nutrition and growth being carried on by osmosis, from cell to cell.

These cells or cavities are supposed by some authors to be the points where ossification begins, as the primary ossific process, then the spider legs begin to be formed, and true bone, but can not exist in the formation of dentine. The formation of haversian canals, the entrance of blood vessels and sinews, being also secondary processes, secondary bone formation being somewhat on a different plan, the final result being the same in plan and construction, first by provisional colloid or white fibrous tissue cells being improvised, mainly from periosteum, then ossification.

The author says further, "We will therefore be justified in the conclusion that there are two kinds of tubes in that part of the dentine nearest to the pulp, one part which receives the processes of the superficial cells, and another to receive the minute nerve fibrils." What data the author finds to base any such conclusion upon, is beyond my comprehension. If he has any good and valid reasons for such conclusions, he certainly has not given them in his paper referred to in this article.

Let him or any one else examine specimens of properly prepared dentine, and he will discover no characteristic differences whatever in the tubuli, no difference in structural outlines, any one being a prototype of all the balance.

Every tube arising in the crown and neck, that have their terminus under the enamel, are continuous from pulp cavity to the base of the enamel, including in this statement all branchings.

I again reiterate the statement, that whatever the character of the tubular contents, they are beyond any doubt whatever, all of the same precise nature, there being no recognizable microscopic difference in dentinal tubes, any theoretical opinions to the contrary notwithstanding.

I believe I am the first writer that ever made the positive statement, *based upon microscopic evidence*, that the nerve fibrils of the pulp, pass out through the pulp membrane, into the tubuli and to their terminations, and gave to the profession my method of investigations, and their numbers into millions, to which objections have been raised by some high in authority.

Nature always having an object in shaping all her means and purposes to ends, doing nothing without an object, certainly would not provide two sets of tubes in the dentine, where only one set was needed, there being no use whatever for any other fibrils in the dentine, except true nerve fibrils.

It seems very evident to my mind, that the author's knowledge of true anatomical relation of pulps and dentine, is certainly very limited and inaccurate.

He speaks about portions of the outer surface of the pulp adhering to the walls of the pulp cavity, whenever an attempt is made to remove the pulp from the walls when crushed in a vise, and the pieces separated.

So far as any portion of a pulp adhering in patches to the sides, when a human tooth is split open and the pulp removed from the walls, is simply an absurdity; such an occurrence never happening under any circumstances whatever. In attempting to extract a pulp from a tooth in the mouth, sometimes portions may be left in the root or roots, the portion coming away being perfect, membrane and all, not a fragment being torn from the body of the pulp and left behind, to which all dentists can testify, who have experience in this method of removing pulps from teeth. A tooth may be split in two, and the pulps also torn in two and remain in their respective pieces of the split tooth, by the mere fibrils.

When a barbed broach is plunged into the pulp of a single tooth, far up the root, and twisted around and withdrawn, most generally the entire pulp comes away with it; sometimes, however, only fragments come away, adherent to the barbs.

This is owing mainly to the fact, that in such cases, the opening to the nerve cavity is not large enough to bring away the pulp entire, or some defect in the broach, or its manipulation from want of experience.

In some of my published articles, several years since, I gave what I conceived to be the true microscopic anatomy of the pulp, and its relation to the dentine, showing conclusively that the pulp had a distinct covering or investing membrane, not however, filling the pulp chamber entirely full, but that there was an appreciable space *by means of a simple magnifier* outside of the pulp membrane, the space being traversed by the myriads of nerve fibrils, in passing through this space from the pulp into the dentinal tubuli, and all interspaces filled with a fluid. It is these nerve fibrils that hold the pulp in situ when a tooth is sawed through or split open. No one fibril from its extreme termination has much strength, but when numbered by millions, their united strength amounts to considerable, when a pulp is removed from the tooth.

In the April number of the *Cosmos*, of 1870, on page 189, the writer says, "These central processes are only distinguished from the lateral ones by their larger size." "Besides these processes already mentioned, there are others in various numbers projecting from the odontoblasts into the tubes of the dentine." "While some cells have only one, I have counted on others three and four, and sometimes even five and six dentinal projections, which gave them the appearance of ciliated cells." "They are distinguished from the two other kinds of processes, by the less granulated protoplasm and brighter lusture." "They branch off and frequently anastomose with each other." "The whole complicated tubular system throughout the dentine, is filled up by these projections."

The author speaks of different kinds of processes of odontoblast passing into the tubuli. Here I would state that these views are simply hypothesis, and not true demonstrations or revelations of the microscope, as all the fibrils that occupy the tubuli are of the same precise character throughout, and as I have for years contended, are genuine, true, sensorial nerve fibrils, and as there can be no possible need or use of any other kind of fibrils in the tubuli, we must ignore the views of the author altogether on that subject of different kinds of tubular fibrils.

"Newman's observations led him to believe that the tooth fibrils become atrophied at the periphery of the teeth of adults." So far, Newman was right, but this atrophied condition applies to the whole extent of the tubes equally perhaps, which however, is the effect of old age, increasing as age advances after middle life has been reached, say 40 years.

The cut on page 192, fig. 4, showing pulp cells, oblong from surface inwardly, with distinctly marked nuclei near their inner, and are in my opinion imaginary, and do not exist. He represents a fibril as coming out from each cell to occupy the tubuli, which is certainly not the microscopic appearance, as his pulp cells are many times larger than the projecting fibrils represented in the cut. The cut may answer well enough as a simple illustration, but as to its being an exact copy as seen under the microscope, I can safely say is not the case.

The author's histogeny of the teeth may be true to a certain extent, but to adopt it as a whole, though mainly the opinions of others would be premature, as we have not yet sufficient data before us at the present time to predicate anything like an exact theory. The idea of the whole pulp mass called odontoblast, cytoblast, or protoplasm, being subject to ossification, and accidentally leaving canals or tubes unossified, leaving a fibril of the same in situ, is certainly erroneous, unless we regard this fibril, as a true nerve fibril, independent of the common odontoblastic matrix, which is the subject of ossification, while the true neural fibrils resist ossification, or in other words, dentification, and when encroached upon in old age, the fibril refuses to undergo ossification, but perishes by absorption first, by gradual closing up around the fibrils, causing their absorption by pressure on their faces.

